# Drought Intensity-Responsive Salicylic Acid and Abscisic Acid Crosstalk with the Sugar Signaling and Metabolic Pathway in *Brassica napus*

**DOI:** 10.3390/plants10030610

**Published:** 2021-03-23

**Authors:** Sang-Hyun Park, Bok-Rye Lee, Van Hien La, Md Al Mamun, Dong-Won Bae, Tae-Hwan Kim

**Affiliations:** 1 Department of Animal Science, Institute of Agricultural Science and Technology, College of Agriculture & Life Science, Chonnam National University, Gwangju 61186, Korea; ghost1284@naver.com (S.-H.P.); turfphy@hotmail.com (B.-R.L.); almamun.uoda@gmail.com (M.A.M.); 2 Department of Biotechnology and Food Technology, Thai Nguyen University of Agriculture and Forestry, Thai Nguten 24000, Vietnam; hiencnsh87@gmail.com; 3 Biomaterial Analytical Laboratory, Central Instruments Facility, Gyeongsang National University, Jinju 52828, Korea; bdwon@gnu.ac.kr

**Keywords:** abscisic acid, ^13^C tracing, drought intensity, hormonal crosstalk, salicylic acid, sugar metabolism

## Abstract

The aim of this study was to characterize hormonal crosstalk with the sugar signaling and metabolic pathway based on a time course analysis of drought intensity. Drought intensity-responsive changes in the assimilation of newly fixed carbon (C) into soluble sugar, the content of sugar and starch, and expression of genes involved in carbohydrate metabolism were interpreted as being linked to endogenous abscisic acid (ABA) and salicylic acid (SA) levels and their signaling genes. The ABA and SA levels in the drought-stressed leaves increased together during the early drought period (days 0–6), and additional ABA accumulation occurred with depressed SA during the late period (days 6–14). Although drought treatment decreased the assimilation of newly fixed C into soluble sugar, representing a 59.9%, 33.1%, and 62.9% reduction in ^13^C-glucose, ^13^C-fructose, and ^13^C-sucrose on day 14, respectively, the drought-responsive accumulation of soluble sugars was significant. During the early period, the drought-responsive accumulation of hexose and sucrose was concurrent with the upregulated expression of *hexokinase 1* (*HXK1)*, which, in turn, occurred parallel to the upregulation of ABA synthesis gene *9-sis-epoxycarotenoid dioxygenase* (*NCED3*) and SA-related genes (*isochorismate synthase 1* (*ICS1*) and *non-expressor of pathogenesis-related gene* (*NPR1*)). During the late period, hexose accumulation, sucrose phloem loading, and starch degradation were dominant, with a highly enhanced expression of the starch degradation-related genes *β-amylase 1* (*BAM1*) and *α-amylase 3* (*AMY3*), which were concomitant with the parallel enhancement of *sucrose non-fermenting−1 (Snf1)-related protein kinase 2* (*SnRK2*)*.2* and *ABA-responsive element binding 2* (*AREB2*) expression in an ABA-dependent manner. These results indicate that the drought-responsive accumulation of sugars (especially SA-mediated sucrose accumulation) is part of the acclamatory process during the early period. Conversely, ABA-responsive hexose accumulation and sucrose phloem loading represent severe drought symptoms during the late drought period.

## 1. Introduction

The dehydration stress-induced decrease in leaf water potential is responsible for the reduction of photosynthesis and C assimilation [1,2]. The decrease in the rate of photosynthesis is attributed mainly to stomatal closure, which is the earliest response to water deficit stress [3]. Decreased photosynthetic CO_2_ assimilation rates under drought conditions [1], followed by the inhibition of de novo amino acid and protein synthesis [4,5], leads to an alteration in the carbohydrate pool [1,6,7]. The pool size of sugars under drought stress is determined by hexose biosynthesis via hexokinase phosphorylation activity [8], sucrose synthesis catalyzed by sucrose phosphate synthase, and sucrose hydrolysis catalyzed by intracellular invertase [8], as well as starch degradation and sugar transport [1,9]. Further, the drought-induced accumulation of soluble sugars has often been observed in previous studies [1,6,7,10]; however, the photosynthesis and carbon assimilation to hexose and sucrose are notably decreased [2,11]. The stress-induced accumulation of soluble sugars plays a role as an osmoprotectant by maintaining the turgor pressure with adequate hydration during water loss [7,10,12]. Sugars, especially sucrose, are important regulatory signals of stress responses and tolerance mechanisms with extensive interactions between sugars and plant hormones; for example, the sugar involvement in the regulation of hormone-responsive pathways [13,14] and, conversely, the hormone-mediated sugar accumulation and sucrose metabolism [7,15,16], as well as the hormonal regulation of leaf starch degradation [7,14,17] and sucrose transport [7,13].

Phytohormone abscisic acid (ABA) accumulation is one of the most rapid responses to abiotic stress, especially drought stress, which causes ABA-induced gene expression [18] and stomatal closure with a subsequent decrease in transpiration and water loss [19], which causes a decrease in photosynthetic activity [20]. A vital role of H_2_O_2_ in ABA-induced stomatal closure is the successive increase in the cytoplasmic Ca^2+^ level, which induces the expression of ABA-responsive genes [21]. Salicylic acid (SA) is also a well-known signaling molecule in abiotic stress tolerance [6,22] and local- and systemic-acquired resistance against pathogens [23,24]. The adverse effects of SA on photosynthetic activity have also been reported, as shown by SA-induced stomatal closure [25,26], SA-decreased Rubisco content [27], and SA-decreased CO_2_ assimilation [28]. Moreover, drought-induced reactive oxygen species (ROS) generation and the elevation of the Ca^2+^ level in leaves are also responsible for SA-induced stomatal closure [10,29].

However, the responses of photosynthetic C assimilation and starch degradation to drought intensity have not been clearly defined regarding the altered hormonal status. Many questions still remain unresolved regarding the complex interconnected signaling and metabolic pathway between a hormone and sugar, which is often associated with the discrepancies observed in their regulatory roles in the stress responses and tolerance mechanism. The present study was designed to (1) directly quantify the de novo synthesis of hexose and sucrose by ^13^C tracing, (2) determine the changes in the carbohydrate metabolism with the possible alteration of endogenous hormonal status, and (3) investigate the signaling and metabolic interactions between a hormone and sugar in the drought-stress responses based on a time course analysis of the drought intensity.

## 2. Results

### 2.1. Leaf Water Status and Physiological Parameters

**Figure 1:**
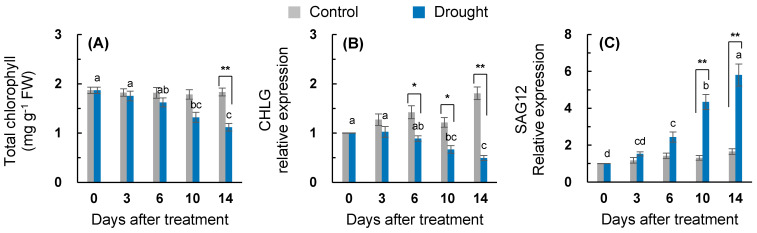
Changes in the (**A**) chlorophyll content, (**B**) *chlorophyll synthesis gene CHLG*, and (**C**) *senescence-associated gene 12* (*SAG12*) in the leaves of the well-watered control or drought-stressed plants during 14 days of treatment. Data are presented as mean ± standard error (SE) for *n* = 4. Bars labeled with different letters in the drought-stressed plants are significantly different at *p* < 0.05, according to Duncan’s multiple range test. Asterisks denote a statistical difference between the drought treatment and control: * *p* < 0.05, ** *p* < 0.01.

The drought treatment was created by decreasing the daily irrigation volume gradually decreased the leaf osmotic potential, relative water content, and leaf dry mass (Table 1). A drought-induced decrease (*p* < 0.05) in the leaf water parameters was apparent after 10 days of treatment. The drought treatment over 14 days decreased the leaf dry mass by 37.7%, when compared to that of the control (Table 1). The chlorophyll content in the drought-stressed leaves was 61% lower than that of the control on day 14 (Figure 1A). The expression of the *chlorophyll synthesis gene* (*CHLG*) was slowly downregulated with the drought stress progression. A significant downregulation of *CHLG* expression occurred after six days of drought treatment (Figure 1B). The expression level of the senescence-related gene *senescence-associated gene 12* (*SAG12*) increased continuously with drought stress progression (+5.8-fold on day 14; Figure 1C).

### 2.2. Endogenous SA and ABA Status

Drought intensity-responsive changes in endogenous the SA and ABA statuses are presented in Table 2. The endogenous SA level in the drought-stressed leaves increased during the early period (days 0–6, a 2.6-fold increase compared to that of the well-watered control leaves) and then significantly decreased to the control levels. The endogenous ABA level in the drought-stressed treatment gradually increased throughout the experimental period, representing a 10.2-fold higher level than that of the control on day 14, whereas no significant change was observed in the control. A drought-responsive increase in the ratio of ABA/SA was distinct after 10 days of treatment.

### 2.3. ABA, SA Synthesis, and Signaling Genes Expression

**Figure 2:**
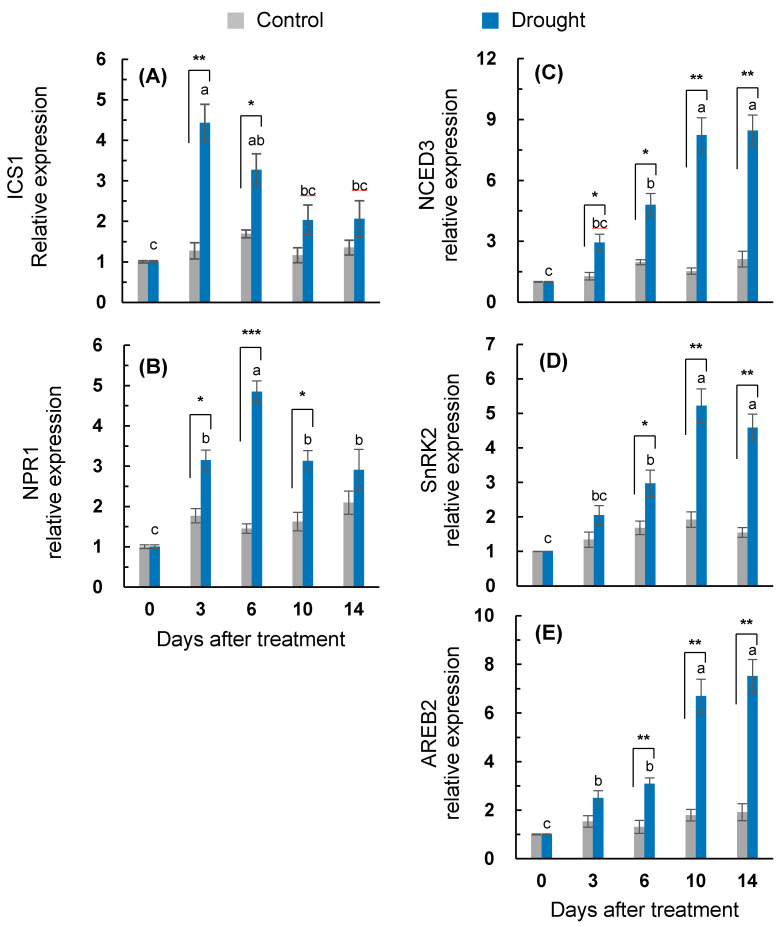
Changes in the expression of the (**A**) salicylic acid (SA) synthesis-related gene *isochorismate synthase 1* (*ICS1*), (**B**) SA-signaling gene, *non-expressor of pathogenesis-related gene*(*NPR1)*, (**C**) ABA synthesis-related gene *9-sis-epoxycarotenoid dioxygenase* (*NCED3*), (**D**) *sucrose non-fermenting−1 (Snf1)-related protein kinase 2* (*SnRK2*), and (**E**) *ABA-responsive element binding 2* (*AREB2*) in the leaves of the well-watered control or drought-stressed plants during 14 days of treatment. Data are presented as the mean ± SE from duplicates for each of the four independent plants. Bars labeled with different letters in the drought-stressed plants are significantly different at *p* < 0.05, according to Duncan’s multiple range test. Asterisks denote a statistical difference between the drought treatment and control: * *p* < 0.05, ** *p* < 0.01, and *** *p* < 0.001.

The expression of a gene associated with SA synthesis, *isochorismate synthase 1* (*ICS1*), in the drought-stressed leaves was highly enhanced during the early period (days 0–6) (Figure 2A). The drought treatment enhanced the expression of the SA-signaling regulatory gene (*non-expressor of pathogenesis-related gene* (*NPR1*) up to day six, as shown with a peak with a 4.9-fold expression compared to the control (Figure 2B). The expression of the ABA synthesis-related gene, *9-sis-epoxycarotenoid dioxygenase* (*NCED3*), in the drought-stressed plants was upregulated throughout the entire treatment period with the most distinct enhancement after 10 days (8.3-fold increase) (Figure 2C). The drought-responsive enhancement of *sucrose non-fermenting−1 (Snf1)-related protein kinase 2* (*SnRK2*) was significant after six days, representing the highest expression on day 10 (5.2-fold increase) (Figure 2D). A drought-responsive upregulation for the ABA-signaling gene *ABA-responsive element binding 2* (*AREB2*) was also significant after six days of treatment, with a stronger activation after 10 days (Figure 2E).

### 2.4. Assimilation of the Newly Fixed ^13^C into Soluble Sugar

**Figure 3:**
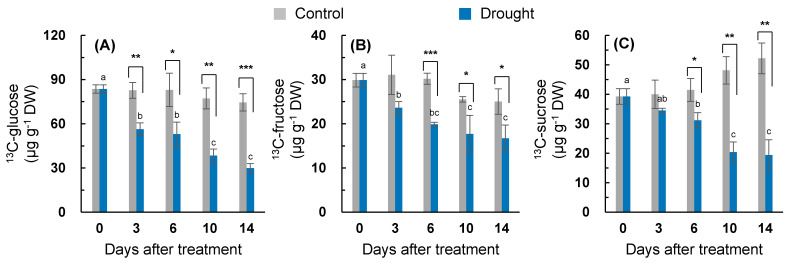
Changes in the amount of newly fixed C assimilated into (**A**) glucose (^13^C-glucose), (**B**) fructose (^13^C-fructose), and (**C**) sucrose (^13^C-sucrose) in the leaves of the well-watered control or drought-stressed plants during 14 days of treatment. Data are presented as the mean ± SE for *n* = 4. Bars labeled with different letters in the drought-stressed plants are significantly different at *p* < 0.05, according to Duncan’s multiple range test. Asterisks denote a statistical difference between the drought treatment and control: * *p* < 0.05, ** *p* < 0.01, and *** *p* < 0.001.

The effect of the drought treatment on the assimilation of the newly fixed C into soluble sugar was estimated by the amount of the recently fixed C incorporated into the glucose (^13^C-glucose; Figure 3A), fructose (^13^C-fructose; Figure 3B), and sucrose (^13^C-sucrose; Figure 3C) fractions. The ^13^C-glucose was less changed within a range of 74.5–83.6 µg g^−1^ dry weight (DW) in the well-watered control. The drought treatment significantly decreased ^13^C-glucose from day three (−31.8%) to day 14 (−60.0%; Figure 3A). A significant decrease in the ^13^C-fructose was also observed from six days after treatment (30.6–34.0% lower than that of the control; Figure 3B). ^13^C-sucrose was slightly increased within a range of 39.2–52.2 µg g^−1^ DW in the control (Figure 3C). Significant drought-responsive decreases in ^13^C-sucrose were observed from day six, and the rate of decrease was enhanced with the progression of the drought intensity (e.g., 13.8% on day three to 60.8% on day 14).

### 2.5. Soluble Sugar and Starch Content

**Figure 4:**
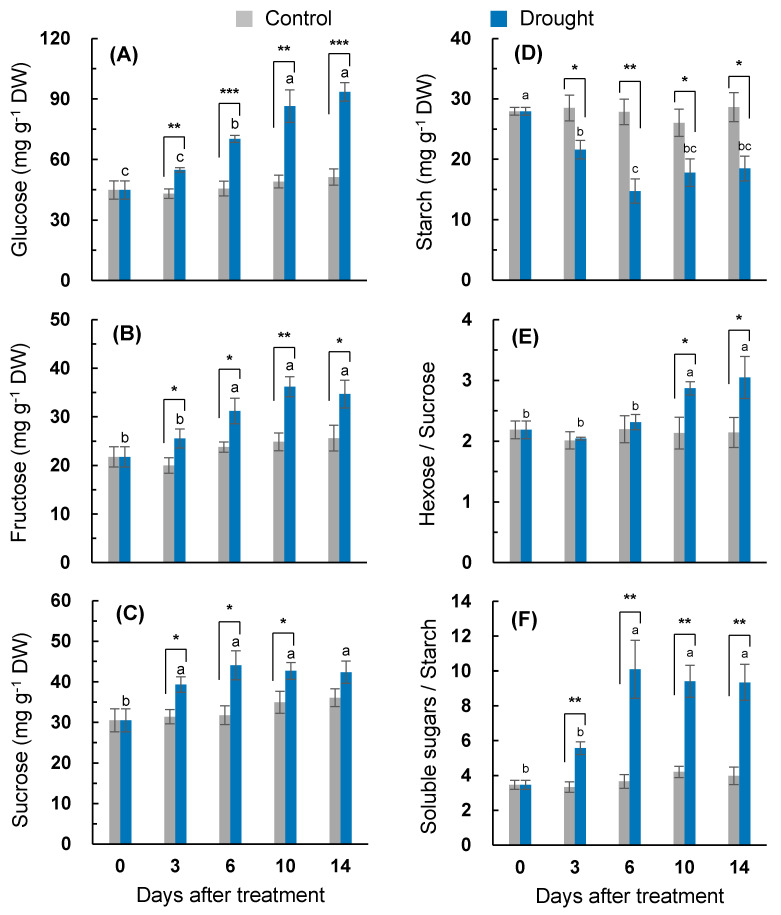
Changes in the (**A**) glucose, (**B**) fructose, (**C**) sucrose, (**D**) starch content, (**E**) hexose/sucrose ratio, and (**F**) soluble sugar/starch ratio in the leaves of the well-watered control or drought-stressed plants during 14 days of treatment. Data are presented as the mean ± SE for *n* = 4. Bars labeled with different letters in the drought-stressed plants are significantly different at *p* < 0.05, according to Duncan’s multiple range test. Asterisks denote a statistical difference between the drought treatment and control: * *p* < 0.05, ** *p* < 0.01, and *** *p* < 0.001.

Changes in the content of the soluble carbohydrate fractions affected by the drought treatment are shown in Figure 4A–C. The glucose content in the drought-stressed leaves significantly increased to day 10, whereas it was less varied within the range of 43.1–51.3 mg g^−1^ DW in the control. The drought-responsive accumulation of glucose was much higher from 10 days in the drought treatment (Figure 4A). The drought-induced accumulation of fructose was significant throughout the experimental period (Figure 4B). The sucrose content increased significantly up to 44.1 mg g^−1^ DW on day six and then stayed stable in the drought-stressed leaves, whereas it remained in the range of 30.5–36.1 mg g^−1^ DW in the control. The drought-induced sucrose accumulation peaked on day six (38.7% higher than that of the control) (Figure 4C). The starch content in the control leaves varied in a range 26.1–28.6 mg g^−1^ DW, significantly (*p* < 0.05) decreased to the lowest level (14.7 mg g^−1^ DW) on day six, and then stayed stable. A drought-induced decrease in the starch content was the highest on day six (47.1% lower than that of the control) (Figure 4D). The resulting ratio of hexose/sucrose was not significantly affected by the drought treatment, remaining within the range of 2.0–2.3 during the earliest six days and then increasing to 3.05 on day 14 in the drought-stressed leaves (Figure 4E). The ratio of soluble sugars/starch also increased in the drought treatment, especially from day six (more than 2.3-fold higher than that of the control) (Figure 4F).

### 2.6. Sugar Metabolism- and Starch Degradation-Related Gene Expression

**Figure 5:**
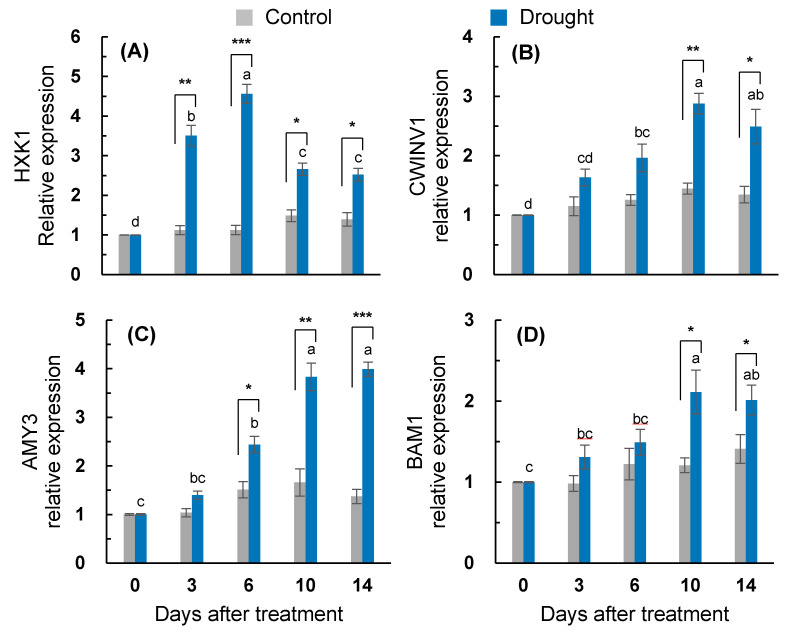
Changes in the relative gene expression of (**A**) *hexose kinase 1* (*HXK1*), (**B**) *cell wall invertase 1* (*CWINV1*), and starch degradation-related genes (**C**) *α-amylase 3* (*AMY3*) and (**D**) *β-amylase 1* (*BAM1*) in the leaves of the well-watered control or drought-stressed plants during 14 days of treatment. Data are presented as the mean ± SE from duplicates for each of the four independent plants. Bars labeled with different letters in the drought-stressed plants are significantly different at *p* < 0.05, according to Duncan’s multiple range test. Asterisks denote a statistical difference between the drought treatment and control: * *p* < 0.05, ** *p* < 0.01, and *** *p* < 0.001.

The drought-responsive enhancement of the *hexokinase 1* (*HXK1*) expression was significant throughout the entire treatment period. The drought-enhanced *HXK1* expression was more distinct for the earliest six days, with the highest expression on day six (4.6-fold increase) and a depression afterward (Figure 5A). The expression of the *cell wall invertase gene* (*CWINV1*) in the drought-stressed leaves was also upregulated; however, its drought-responsive enhancement was significant only on days 10 and 14 (Figure 5B). The drought treatment enhanced the expression of the starch degradation-related gene *α-amylase 3* (*AMY3*) from six days after treatment and showed the highest expression on day 14 (4.2-fold increase; Figure 5C). The drought-responsive enhancement of *β-amylase 1* (*BAM1*) expression was significant after 10 days of treatment (2.1-fold increase; Figure 5D).

### 2.7. Sucrose Transport and Its Contribution to Osmotic Adjustment

**Figure 6:**
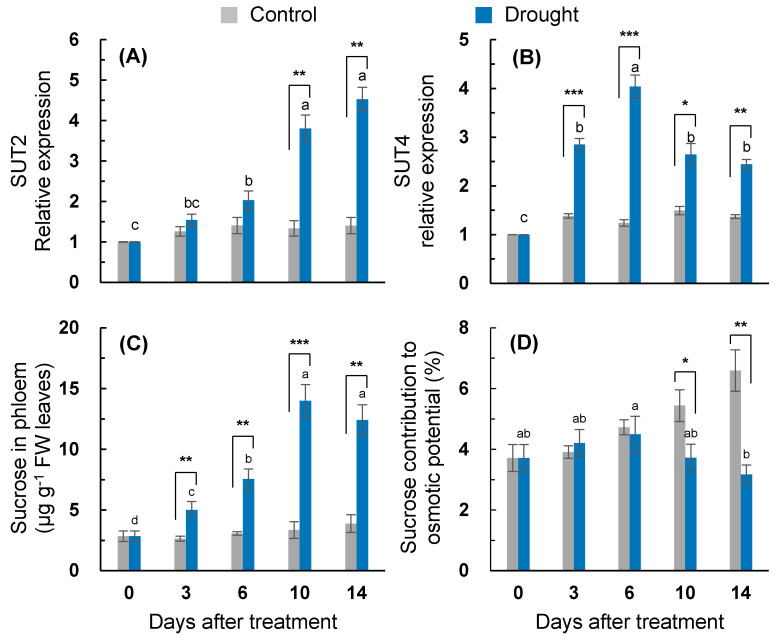
Changes in the relative gene expression of sucrose transporters (**A**) *SUT2* and (**B**) *SUT4*, (**C**) sucrose content in the phloem exudate, and (**D**) contribution percentage of the sucrose-to-osmotic potential in the leaves of the well-watered control or drought-stressed plants during 14 days of treatment. Data are presented as the mean ± SE from duplicates for each of the four independent plants. Bars labeled with different letters in the drought-stressed plants are significantly different at *p* < 0.05, according to Duncan’s multiple range test. Asterisks denote a statistical difference between the drought treatment and control: * *p* < 0.05, ** *p* < 0.01, and *** *p* < 0.001.

Among the four sucrose transporter genes (*SUT1, SUT2, SUT4,* and *SWEET11*) examined during the present study, the expression of *SUT2* and *SUT4* were distinctly expressed in response to drought stress (Figure 6A,B), while *SUT1* and *SWEET 11* were less responsive (Supplementary Figure S1). The drought treatment tended to enhance the expression of the sucrose transporter. The drought-induced enhancement of *SUT2* expression was much higher after 10 days of treatment. The highest expression of *SUT2* was recorded on day 14 (4.6-fold increase) (Figure 6A). The expression of *SUT4* in the drought-stressed leaves peaked on day six (4.0-fold increase) and then significantly depressed (Figure 6B). The sucrose content in the phloem exudate was significantly increased by the drought treatment throughout the entire experimental period, with the highest content found on day 10 (4.2-fold higher than that of the control) (Figure 6C). The contribution percentage of the sucrose-to-osmotic potential in the control tended to increase, whereas in the drought-stressed leaves, it significantly decreased from day 10 (Figure 6D).

### 2.8. Correlation among Variables Measured Parameters, Hormones, and Their Regulated Genes Expression for the Early and Late Phases of Drought Stress

**Figure 7:**
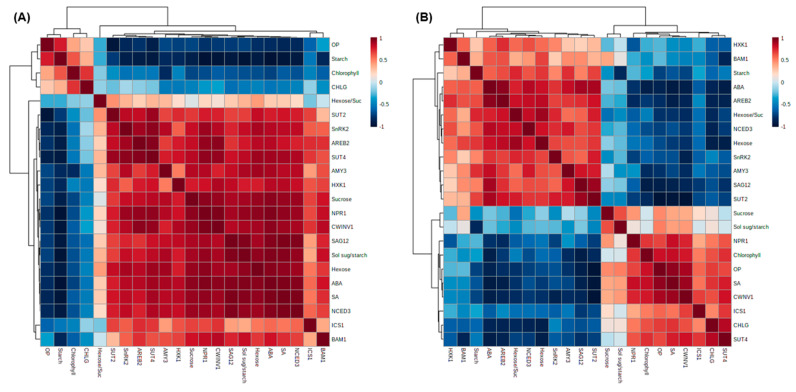
Heatmap showing the correlations among the identified hormones, metabolites, and gene expression during the early period (days 0–6; (**A**)) and late period (days 6–14; (**B**)). The correlation coefficients were calculated based on Pearson’s correlation. Red indicates positive effects, whereas blue indicates negative effects. The color intensity is proportional to the correlation coefficients. Suc: sucrose and Sol sug: soluble sugars.

To further investigate the physiological relationship between the measured variables through the drought stress intensity, we used the Pearson correlation coefficients among the descriptive parameters of the hormonal status, leaf senescence, and sugar metabolism (Figure 7). For the early phase (days 0–6), the endogenous SA level, in the close, positive correlations with *ICS1*, *NPR1*, and *HXK1* expression, was shown to closely relate with ABA and its signaling-related genes, sucrose and hexose contents, and sucrose transporter genes *SUT2* and *SUT4* expression. Negative correlations were observed with the osmotic potential, starch, and chlorophyll contents (Figure 7A). For the late phase (days 6–14), ABA, which was positively correlated with *NCED3*, *SnRK2*, *AREB2*, and the starch degradation-related genes *AMY3* and *BAM1* expression, was negatively correlated with the SA level and SA-regulated genes *ICS1* and *NPR1* expression and sucrose transporter gene *SUT4* and *CWINV1* expression (Figure 7B).

## 3. Discussion

### 3.1. Carbohydrate Metabolism in Response to Drought-Stress Intensity

By decreasing the daily irrigation volume over 14 days, the drought treatment successfully induced dehydration stress in *Brassica napus* leaves, as shown by the reduction in leaf osmotic potential (40% decrease) and leaf biomass (34% decrease), compared to those of the well-watered control plants (Table 1). A decrease in the leaf water parameter, caused by hydraulic or osmotic stress, is responsible for decreasing the stomatal conductance, photosynthesis, and carbon and nitrogen assimilation [1,2,4,30]. A drought-responsive decline in the internal CO_2_/O_2_ ratio attributed to stomatal closure enhances photorespiration and ROS production, leading to lipid peroxidation and the oxidation of proteins and DNA/RNA molecules during oxidative stress [31], which is responsible for the disruption of photosynthetic carbon assimilation and nitrogen uptake and assimilation [1,4,5,30]. As one of the earliest responses to drought stress, a decrease in the photosynthesis rate paralleled with a decreased stomatal conductance results in a reduction in the photosynthetic CO_2_ assimilation [1]. The assimilation of the newly fixed ^13^C into glucose, fructose, and sucrose was significantly decreased within the first six days of drought treatment (Figure 3), although the leaf osmotic potential and relative water content were not significantly decreased up to day six (Table 1). In the present study, it was estimated that the drought treatment for 14 days decreased the amount of ^13^C-glucose, ^13^C-fructose, and ^13^C-sucrose by 59.9%, 33.1%, and 62.9%, respectively (Figure 3). Previous studies have shown an approximate 20% decrease in photosynthetic ^14^C-CO_2_ assimilation to sucrose in barley leaves exposed to osmotic stress [32] and a 52% decrease in the amount of newly fixed ^13^C incorporated into soluble sugars in white clover leaves exposed to drought stress for seven days [1].

The drought intensity has an influence on the balance between photosynthetic C fixation and the partitioning of photoassimilates in sinks, leading to an alteration in the magnitude of carbohydrate pools. The accumulation of soluble sugars, which has been widely reported during water loss [1,3,6,7,32], was confirmed under the drought conditions in the present study. A drought-responsive sucrose accumulation was significant from days three to six (Figure 4C), leading to the higher enhancement of *CWINV1* expression (Figure 5B), whereas the accumulation of glucose and fructose was more distinct during the late period (days 6–14) (Figure 4A,B), with a highly enhanced expression of *HXK1* (Figure 5A). Drought-responsive starch depletion occurred from three days after the start of the treatment (Figure 4D). The drought-enhanced expression of starch degradation-related genes (*AMY3* and *BAM1*) was more distinct during the late period (days 6–14) (Figure 5C,D). Given that the drought-responsive decrease in the newly fixed ^13^C assimilation into glucose, fructose, and sucrose was significant, at the latest, from day six (Figure 3), the accumulation of soluble sugars under drought conditions was mainly caused by the starch degradation rather than by the de novo synthesis of hexose and sucrose. The starch degradation and soluble sugar accumulation in various plants in response to dehydration stress have also been observed [1,7,32]. The resulting ratio of soluble sugars/starch was significantly increased throughout the entire drought treatment in the present study (Figure 4). Therefore, the starch pool in the drought-stressed plants was depleted and the resulting accumulation of hexose and sucrose might be involved in the observed feedback inhibition of photosynthesis and the newly fixed C assimilation [1,12]. The drought-responsive sucrose accumulation was relatively higher during the early period (days 3-6) (Figure 4C), whereas the hexose accumulation was higher during the late period (days 6–14) (Figure 4A,B), resulting in a significant increase in the ratio of hexose/sucrose only for the late period (days 10–14) (Figure 4E). Underlining the overall agreement that the stress-induced accumulation of soluble sugars has a positive role in stabilizing cellular membranes and maintaining their turgor [32], we estimated the contribution percentage of soluble sugars to the osmotic potential. The sucrose contribution percentage to the osmotic potential in the drought-stressed plants was similar to that in the control during the first six days (Figure 6D), whereas the hexose contribution was much lower in the drought-stressed plants throughout the entire drought period (data not shown). Therefore, drought-responsive sucrose accumulation, which occurred from days three to 10, was involved in adjusting the leaf osmotic potential during the first six days of treatment (Table 1). The drought-responsive enhancement of the sucrose phloem loading was more prominent during the late period (days 10–14) (Figure 6C) with the enhanced expression of sucrose transporter genes *SUT2* and *SUT4* (Figure 6A,B), which are involved in the sucrose phloem loading [9,14].

The data from the present study indicate that the drought-induced reduction in the *de novo* synthesis of soluble sugar was accompanied by a compensable sucrose accumulation during the first six days and hexose accumulation derived mainly from starch degradation during the late period. Drought stress-induced changes in the endogenous hormonal status and signaling pathways play an important role in regulating the stress responses and tolerance mechanism [6,7,33,34]. Therefore, in the present study, the drought-induced alteration in the sugar metabolism was evaluated in relation to specific hormonal changes and in the expression of the regulatory genes in terms of the drought responses and tolerance based on a time course analysis of drought intensity.

### 3.2. SA-Mediated Acclimation during the Early Period of Drought Stress

In the present study, during the early period (days 0–6), a drought-responsive increase in the endogenous ABA and SA levels (Table 2) was accompanied by a significant reduction in the newly fixed ^13^C assimilation to glucose, fructose, and sucrose in the drought-stressed leaves (Figure 3). In our previous study with white clover, a water deficit-induced decrease in the leaf water potential occurred in parallel with a reduction in the net photosynthesis rate and stomatal conductance, leading to a positive correlation between the decrease in the leaf water potential and the decreased ^13^C assimilation to soluble sugars [1]. Numerous studies have shown that stomatal closure, which is the main cause of decreased photosynthesis under drought conditions, is mediated by hormone control [3,18]. The stress-induced ABA enhances the respiratory burst oxidase homolog expression, resulting in H_2_O_2_ production, which triggers the activation of ABA signaling [35]. This feed-forward loop between ABA and H_2_O_2_ activates the plasma membrane Ca^2+^ channels, leading to an increase in cytosolic Ca^2+^ in the guard cells [20,21], thereby inducing ABA-mediated stomatal closure [36] and consecutively reducing the photosynthetic activity [20]. In addition to ABA, stress-responsive SA accumulation is broadly accepted as the key signal for stress responses and the resistance mechanism [22,29]. Stomatal closure in response to an external SA application has also been observed [25,26,37]. A previous study showed that constitutively high levels of SA-decreased stomatal conductance led to a decrease in the maximum efficiency of photosystem II [38].

However, existing controversy means that the effect of SA on photosynthesis remains to be determined. SA is tightly linked to the redox homeostasis and photosynthetic performance [6,38]. In addition, it has been shown that SA application alleviates the stress-induced decrease in photosynthesis [39,40] by enhancing the nitrogen and sulfur assimilation and antioxidant metabolism [40] or increasing the enzyme activity involving CO_2_ uptake rather than increased stomatal conductance [39]. Mateo et al. [38] reported that the SA levels are required to be controlled to maintain the optimal photosynthesis and redox homeostasis. The chlorophyll degradation and leaf senescence mediated by ABA in response to drought stress has been alleviated to the control level in SA-pretreated plants, accompanied with the improvement of the endogenous SA level and PR2 expression [6]. Similarly, during the first six days when drought SA accumulation alone occurred (Table 2), there were no significant changes in the leaf osmotic potential, relative water content (Table 1), chlorophyll content (Figure 1A), or *SAG12* expression (Figure 1C), although the drought-responsive reduction in the assimilation of newly fixed ^13^C into glucose, fructose, and sucrose was significant (Figure 3). Therefore, the drought-induced chlorophyll degradation and leaf senescence are alleviated by SA during mild water limitation.

Positive effects of SA on sugar accumulation have been widely reported [7,10]. In the present study, the drought-responsive sucrose accumulation was much higher during the early period (Figure 4C). The sucrose accumulation during this period was responsive to the increased expression of *HXK1.1* (Figure 5A) in parallel with the expression of SA-regulated genes *ICS1* and *NPR1* (Figure 2A,B and Figure 7A). Hexose kinase, as a glucose sensor, phosphorylates glucose or fructose during sucrose synthesis and storage [41]. In our previous study, the drought-induced sucrose accumulation was additionally increased by the enhanced SA level and the activated SA-regulated gene *PR2* in the SA-pretreated drought-stressed plants, thereby significantly alleviating the decrease in osmotic potential and leaf senescence [7]. In the present study, in the first six days, SA-mediated sucrose accumulation was accompanied by significant increases in phloem sucrose loading (Figure 6C) with an enhanced expression of the sucrose transporter gene *SUT4* (Figure 6B), thereby representing a similar sucrose contribution to the osmotic potential as that of the control (Figure 6D). Therefore, during the early period of drought stress (days 0–6), SA-mediated sucrose accumulation is part of the acclamatory resistance process for regulating the osmotic potential and leaf senescence (Figure 7A).

### 3.3. ABA-Mediated Sugar Signaling and Drought Symptom Development during the Late Period

During the late drought phase (days 6–14), stress symptomatic responses (e.g., the lowest osmotic potential, chlorophyll content (Figure 1A), ^13^C assimilation to soluble sugars (Figure 3), and the loss of the leaf dry mass (Table 1)) were obviously developed with the increasing ABA levels (Table 2). However, the SA level and SA-related gene (*ICS1* and *NPR1*) expression were largely repressed (Figure 2A,B). The resulting ratio of ABA/SA highly increased from day 10 in the drought-stressed leaves, which was 12.6-fold higher than that of the control (Table 2). ABA-signaling activation in stressed plants is attributed to stress-induced ROS, especially to H_2_O_2_ signaling through the activation of respiratory burst oxidase homolog expression [35], which, in turn, leads to an activation of ABA responses via ABA synthesis [36]. The time course analysis in *Arabidopsis* showed that the crosstalk between H_2_O_2_ and ABA has a much later peak time than that of SA [42]. In the present study, SA accumulation had a peak on day six, whereas the highest endogenous ABA level was observed on day 14 (Table 2). Consistent with ABA accumulation during the late drought period (days 6–14), the dominant enhancement of ABA synthesis, 9-sis-epoxycarotenoid dioxygenase (*NCED3*), and ABA-responsive element bind 2 (*AREB 2*) expression were observed (Figure 2C,E). These ABA responses showed a parallel enhancement of senescence-related gene (*SAG12*) (Figure 1C), accompanied by a decreased chlorophyll content (Figure 1A), with a depressed expression of chlorophyll synthase-related gene (*CHLG*; Figure 1B). Several studies have shown that ABA accelerated the chlorophyll degradation as a positive regulator of the leaf senescence [7,14], and the ABA-responsive elements (*AREBs*) act as key regulators in mediating ABA-triggered chlorophyll degradation and leaf senescence [43]. Therefore, drought-induced ABA signaling was involved in modulating the chlorophyll degradation and leaf senescence, especially during the late drought period when the SA level and expression of SA-related genes were depressed (Figure 7B).

In the present study, during the late period (days 6–14), the drought-induced ABA-responsive sugar metabolism was characterized by an accumulation of hexose (glucose + fructose), which resulted in increases in the hexose/sucrose ratio (Figure 4E) and starch degradation, leading to increases in the soluble sugar/starch ratio (Figure 4D,F). Starch degradation with a highly enhanced expression of starch degradation-related genes (*AMY3* and *BAM1*) (Figure 5C,D) was concomitant with the parallel enhancement of *NCED3* and *AREB 2* (Figure 2C,E) in an ABA-dependent manner (Figure 7B). The expression of *sucrose non-fermenting−1 (Snf1)-related protein kinase 2* (*SnRK2*), which is required for ABA synthesis and signal transduction [15,16], was also highly increased (Figure 2D). Similarly, in *Arabidopsis*, ABA-induced *AREB2* activated the expression of α-amylase genes such as *AMY3* and *BAM1* via an ABA-dependent *SnRK2* signaling pathway [17]. Ma et al. [13] reported that the ABA-responsive element-binding transcription factor promoted the accumulation of soluble sugars by directly binding to the starch degradation-related genes *AMY3* and *BAM1* in apples. Moreover, drought-enhanced ABA-responsive hexose accumulation paralleled the enhancement of sucrose phloem loading, especially for the late period (Figure 6C) accompanied by enhanced *SUT2* expression (Figure 6A and Figure 7B). In our previous study, the ABA-responsive *SnRK2*- and *AREB2*-mediated enhancement of sucrose phloem loading was observed in the source leaves during the bolting stage to support sucrose filling in the pod [14]. Therefore, the results presented and those from previous studies [13,15,16,17] suggest that hexose accumulation and sucrose phloem loading during the late period are mainly attributed to the enhanced starch degradation and activation of sucrose transporter gene *SUT2* under the mediation of the ABA-responsive transcription factors *SnRK2* or *AREB2*. The accumulation of soluble sugars via hexose kinase [44] and starch degradation [1,6,32] is associated with stress tolerance by adjusting the osmotic potential in various plants exposed to dehydration stress. However, the enhanced hexose level in an ABA-dependent manner was not consistent with the proposed role in osmotic adjustment, as shown by the decrease in the leaf osmotic potential and dry mass (Table 1) and the highly enhanced expression of *SAG12* (Figure 1C) with visibly severe leaf wilting. These results support the notion that osmotic stress-induced hexose accumulation may be a senescence-associated response [9].

**Figure 8:**
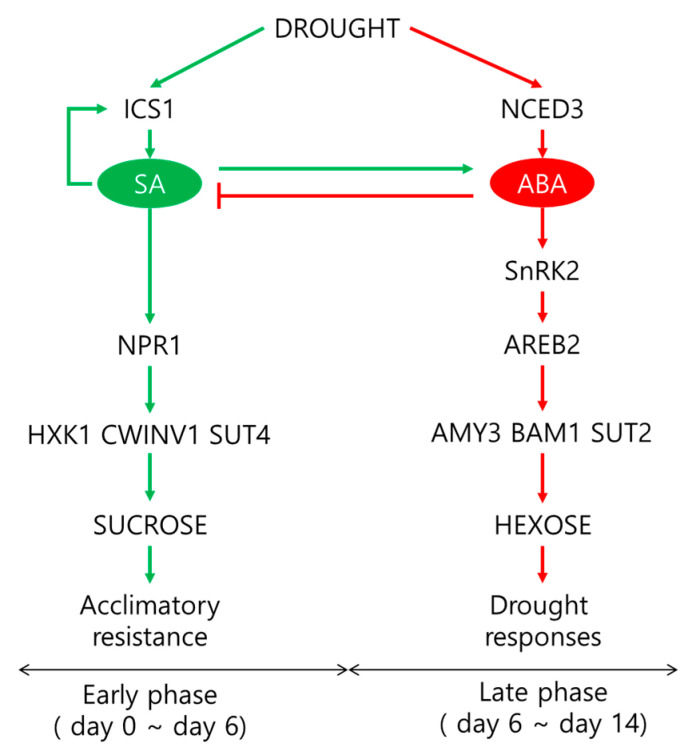
Proposed model of crosstalk between SA, ABA, and the sugar metabolism in two distinct phases of drought intensity. Green and red arrows represent the SA- and ABA-dependent pathways, respectively, in response to drought.

These results confirm the interactions between hormones and their sugar metabolism, which have been widely reported previously [6,7,13,14,15,16,17]. Furthermore, to the best of our knowledge, the present data are the first to report that the drought intensity influences the hormonal crosstalk with sugar metabolic or signaling pathways (Figure 8), representing two distinct phases characterized by (1) a significant involvement of SA-mediated sucrose accumulation via *NPR1* in regulating the osmotic potential and leaf senescence as an acclamatory process under a mild drought stress intensity (days 0–6) and (2) ABA-responsive hexose accumulation and sucrose phloem loading via mainly starch degradation under the mediation of ABA-responsive transcription factors *SnRK2* or *AREB2*, leading to severe drought symptoms during the later period (days 6–14).

## 4. Materials and Methods

### 4.1. Plant Growth, Drought Treatment, and Leaf Water Parameter Measurement

*Brassica napus* (cv. Capitol) seeds were sown in bed soil in a tray. At the 4-leaf stage, the seedlings were transferred to a 2-L pot containing a mixture of soil and perlite (70:30, *w/w*) in a greenhouse. A complete nutrient solution was continuously supplied to the plants [11]. Natural light was supplemented by metal halide lamps, which generated *c.* 400-µmol photons m^−2^s^−1^ at the canopy height for 6 h per day. Plants were selected by morphological similarity after 6 weeks and divided into two groups that were normally irrigated with 200 mL for well-watered plants (control) or 20 mL for drought-stressed plants. Sampling was undertaken at 3, 6, 10, and 14 days after the commencement of the drought treatment. The biomass, ABA and SA contents, gene expression level *ICS1*, *NPR1*, *NCED3* were previously published by Park et al. [45].

Leaf osmotic potential was measured immediately as the petiole xylem pressure potential using a pressure chamber (PMS Instrument Co., Corvallis, OR, USA). Relative water content was determined as described previously [4]. Leaf water status measurements were taken before dawn on the first or second fully expanded green leaf proximal to a stolon apex.

### 4.2. CO_2_ Labeling

The ^13^CO_2_ labeling was undertaken one day before each sampling date. The plants were transferred from the natural atmosphere of the greenhouse to a growth cabinet containing an atmosphere enriched up to 1.278 atom % ^13^C (0.19% over natural abundance). The CO_2_ concentration was maintained at 450 mm^3^ dm^−3^ in the growth chamber with a day/night temperature of 28/20 °C and a photoperiod of 18 h. The ^13^C level of CO_2_ was controlled by analyzing triplicate samples of the atmosphere taken four times per 24 h (at the beginning and end of the light and night periods) through an exit valve using previously evacuated 30-cm^3^ flasks. During labeling and the chase periods, at the beginning and end of each photoperiod, CO_2_-free air (obtained from ambient air compressed to approximately 8 MPa by a screw compressor and passed through a NaOH column) was injected for 20 min into the cabinet to evacuate CO_2_ and decrease the plant reassimilation of ^13^CO_2_. To eliminate the plant isotope discrimination, ^13^CO_2_ exposure was started at the beginning of each light period and stopped 2 h before the light was switched off. Thus, the CO_2_ had disappeared almost totally from the chamber atmosphere by the end of the light period, and all CO_2_ introduced into the chamber during the light period was assimilated [46]. At the end of the labeling period, the assimilation cabinet was opened and quickly purged with ambient air.

### 4.3. Collection of Phloem Exudate and Sucrose Measurement 

Phloem exudates were collected using the facilitated diffusion method with ethylenediaminetetraacetic acid (EDTA), according to the methodology described by reference [30]. The fourth fully extended leaf was cut and immediately immersed in 20-mM EDTA solution (pH 7.0) for 5 min, and then, the EDTA solution was discarded. The leaf was rinsed, transferred to a new tube containing 5-mM EDTA solution, and kept for 6 h in a growth chamber with 95% relative humidity under dark conditions. The sucrose content in the phloem exudate was determined according to the method described by Van Handel [47].

### 4.4. Extraction, Fractionation, and Analysis of Carbohydrates

Soluble carbohydrates were extracted from freeze-dried tissues by first boiling under reflux (1 h) with 80% ethanol, followed by boiling water under reflux (1 h). The extracts were dried under vacuum, combined, and redissolved in water. Aliquots of carbohydrate extracts were passed through a column containing cation exchange resin (Dowex 50 W, H^+^ form, Sigma, St. Louis, MO, USA) and anion exchange resin (Amberlite IRA−416, Flucka, Buchs, Switzerland) to remove the charged compounds. The columns were eluted with water, and the samples were concentrated under vacuum and dissolved in water (total soluble sugar fraction). Glucose, fructose, and sucrose were separated with high-performance liquid chromatography (HPLC) on a cation exchange column (Sugar-PAK, 300 mm × 6.5 mm, Millipore water, Milford, MA, USA) using mannitol as an internal standard. After separation with a Sugar-PAK column, glucose, fructose, and sucrose were collected separately and were then concentrated under vacuum, dried on chromosorb (Europa Scientific, Crewe, UK), and packed in tin capsules until a subsequent analysis with an isotope ratio mass spectrometer (IsoPrime, GV Instrument, Manchester, UK).

The ethanol-extracted residue was dried at 80 °C to remove the ethanol. Deionized water was added and heated to gelatinize the starch. The pH of the solution was adjusted to 5.1 by adding 0.2-N Na-acetate buffer. The starch was digested by adding amyloglucosidase (Sigma product A3514) and α-amylase (Sigma product A0273) to the acetate buffer of each sample. The tubes were incubated at 50 °C for 24 h with occasional shaking and then centrifuged. The glucose in the supernatant was measured with glucose oxidase (Glucose Trinder kit, Sigma product 315–100). Starch concentrations were calculated by multiplying the glucose concentration by a factor of 0.9 [47].

### 4.5. ^13^C Isotope Analysis of Carbohydrate Fractionations

Freeze-dried powder samples (2–3 mg) were weighed into tin capsules for total C determination. The C and ^13^C content of the plant samples and solutions of soluble sugars were quantified with a continuous flow isotope mass spectrometer linked to a C/N analyzer (EA 3000, EuroVector, Milan, Italy). The atmospheric samples of the cabinet were previously purified in a gas analyzer (Roboprep G+, Europa Scientific) before injection into the mass spectrometer. The obtained ^13^C abundances were converted to relative specific activities (RSA, i.e., % of recently incorporated atoms relative to the total numbers of atoms in the sample) using Equation (1): RSA = (^13^C atom % measured – natural ^13^C atom %) **/** (^13^C atom % ^13^CO_2_ supplied – natural ^13^C atom %) × 100 (1), where the natural ^13^C atom % was adopted from the ^13^C atom % of non-^13^C-fed plants. The amounts of the recently fixed ^13^C (RFC) incorporated in the organic compounds were calculated per gram dry weight with Equation (2): RFC = (RSA X C content measured in a compound)/100 (2).

### 4.6. Phytohormone Analysis

Quantitative analysis of SA and ABA in the leaf tissue was performed according to the methodology described by Pan et al. [48]. Phytohormone extracts from 50 mg of freeze-dried leaves were injected into a reverse-phase C18 Gemini HPLC column for HPLC electrospray ionization tandem mass spectrometry analysis. Agilent 1100 HPLC (Agilent Technologies), Waters C18 column (150 × 2.1 mm, 5 µm), and API3000 MSMRM (Applied Biosystems) were used for the analysis.

### 4.7. Isolation of Total RNA and Quantitative Real-Time Polymerase Chain Reaction (qRT-PCR)

Total RNA was isolated from the 200 mg of leaf tissue using RNAiso Plus (Takara, Nojihigashi 7–4−38 Kusatsu, Shiga, Japan). The GoScript Reverse Transcription System (Takara) was used to synthesize cDNA from the RNA. A light cycle real-time PCR detection system was used to quantify the gene expression level. The PCR was initiated at 95 °C for 5 min, followed by 45 cycles of 95 °C for 30 s, 51 °C-60 °C for 30 s (depending on the target primers), and 72 °C for 30 s, with a final extension at 72 °C for 5 min. The qRT-PCRs were performed in duplicate for each of the four independent samples. The sequences of primer are presented in Supplementary Table S1. All the quantifications were normalized to actin.

### 4.8. Statistical Analysis

A completely randomized design was used with three replicates for each treatment. Duncan’s multiple range test was employed to compare the means of the separate replicates. Statistical significance was postulated at *p* < 0.05. Statistical analysis of all measurements was performed using the SAS 9.1.3 software program (SAS Institute Inc., Cary, NC, USA).

## Figures and Tables

**Table 1:** Changes in the leaf osmotic potential, relative water content, soil water content, leaf biomass, and leaf total carbon concentration in the leaves of well-watered (control) or drought-stressed plants for 14 days.

Physiological Parameters	Days after Treatment				
/Treatment	0	3	6	10	14
Leaf osmotic potential (MPa)					
Control	−0.105 ^a^	−1.117 ^a^	−1.178 ^ab^	−1.211 ^ab^	−1.177 ^ab^
Drought	−0.102 ^a^	−1.265 ^ab^	−1.399 ^b^	−1.712 ^c^	−1.950 ^d^
Relative water content (RWC, %)					
Control	86.33 ^ab^	87.32 ^a^	86.56 ^ab^	84.91 ^ab^	85.07 ^ab^
Drought	86.56 ^ab^	82.79 ^ab^	76.38 ^bc^	72.64 ^c^	70.98 ^c^
Leaf biomass (DM, g plant^−1^)					
Control	8.05 ^f^	8.78 ^ef^	11.94 ^bc^	13.40 ^b^	16.01 ^a^
Drought	8.03 ^f^	8.24 ^f^	10.53 ^cd^	9.59 ^def^	9.97 ^de^

Values are means of three replicates with three plants each. Values in a vertical column or a horizontal row followed by different letters are significantly different at *p* < 0.05, according to Duncan’s multiple range test.

**Table 2:** Changes in the endogenous level of abscisic acid (ABA), salicylic acid (SA), and their ratio in the leaves of well-watered (control) or drought-stressed plants during 14 days of treatment.

Days after Treatment	0		3		6		10		14	
Treatment	Control	Drought	Control	Drought	Control	Drought	Control	Drought	Control	Drought
SA (ng g^−1^ DW)	3.65 ^e^	3.65 ^e^	4.39 ^cde^	6.24 ^b^	3.87 ^de^	9.54 ^a^	5.60 ^bc^	6.27 ^b^	5.11 ^bcd^	4.14 ^de^
ABA (ng g^−1^ DW)	6.76 ^e^	6.76 ^e^	7.38 ^e^	21.91 ^d^	8.09 ^e^	38.86 ^c^	10.89 ^e^	83.64 ^b^	9.58 ^e^	97.30 ^a^
ABA/SA ratio	1.86 ^c^	1.86 ^c^	1.71 ^c^	3.55 ^c^	2.10 ^c^	4.06 ^c^	2.01 ^c^	13.52 ^b^	1.92 ^c^	24.04 ^a^

Values in a vertical column or a horizontal row followed by different letters are significantly different at *p* < 0.05 according to Duncan’s multiple range test.

## Data Availability

The data presented in this study are available within the article and its Supplementary Materials.

## Supplementary Materials

The following are available online at https://www.mdpi.com/2223-7747/10/3/610/s1. Figure S1: Changes in the relative gene expression of sucrose transporters (A) SUT1 and (B) SWEET11 in the leaves of the well-watered control or drought-stressed plants during 14 days of treatment. Data are presented as the mean ± SE from duplicates for each of the four independent plants. Bars labeled with different letters in the drought-stressed plants are significantly different at *p* < 0.05, according to Duncan’s multiple range test; Table S1: The specific primers used for qRT-PCR.
