# Supplementary material for: Drought Intensity-Responsive Salicylic Acid and Abscisic Acid Crosstalk with the Sugar Signaling and Metabolic Pathway in Brassica napus
Source: Plants (Basel). 2021 Mar 23;10(3):610. doi: 10.3390/plants10030610 (PMC8004980; doi:10.3390/plants10030610)
Supplement: Supplementary file 1 [file plants-10-00610-s001.pdf]

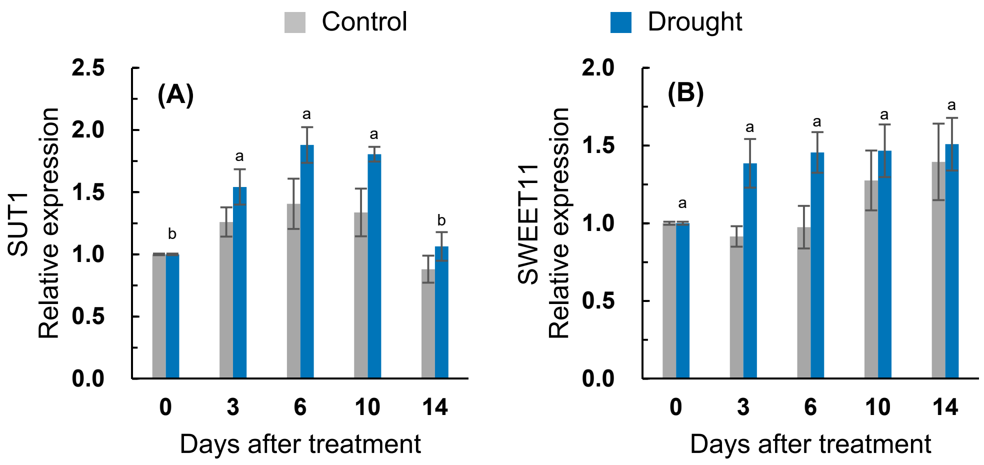

Supplementary Figure S1. Changes in the relative gene expression of sucrose transporters (A) SUT1 and (B) SWEET11 in the leaves of the well-watered control or drought-stressed plants during 14 days of treatment. Data are presented as mean ± SE from duplicate for each of the four independent plants. Bars labeled with different letters in the drought-stressed plants are significantly different at $p < 0.05$ according to Duncan's multiple range test.

Supplementary Table S1. Specific primers used for qRT-PCR.

Target gene	GenBank Number	Forward sequence	Reverse sequence
BnICS1	XM013887885	5′-TCAATCCCAGAACGAGATCC-3′	5′-GACAGAAACCTTCGGATGGA-3′
BnNPR1	EF613226.1	5′-TGAGAACATTGCCAAGCAAG-3′	5′-CAACAGCAAAATGGAGAGCA-3′
BnCHLG	XM013788949.1	5′-CTACGAACTCGTCAC CAAAG-3′	5′-AGGTCCAAACCAATGATTCT-3′
BnNCED3	HQ260434	5′-GGAGTGCTTCTGCTTCCATC-3′	5′-TTCGAGGTTGACTTGCTCCT-3′
BnSAG12	XM013821610.2	5′-AGAGAATACCAAACCAAACCGAA-3′	5′-GCAACTCCCAAAATCTCAGGG-3′
BnSnRK2	LK937699.1	5′-TGAAGATGAGGCTCGGTTCT-3′	5′-TGCCATCATATTCCTGACGA-3′
BnAREB2	HE616526.1	5′-AGATTGCTGCCAAAGATGCT-3′	5′-CACCTCTTATCCCAGGACCA-3′
BnHXK1	XM_013797259.1	5′-TTCTCCGGATTTGAAGGTTG-3′	5′-GTCTTTCGGTGCGTCTCTTC-3′
BnCWINV1	KP872754.1	5′-TCTACCAATGGAACCCCAAA-3′	5′-CGGCTATGTTTTGGACCTGT-3′
BnAMY3	XM013846160.2	5′-GGTTACCTCCACCGACAGAA-3′	5′-GTTCAGACGCCCTCCAAATA-3′
BnBAM1	XM013852497.2	5′-GAAGGTGGGGCTAAAGGTTC-3′	5′-GCACGCATGAAATCAGAGAA-3′
BnSUT1	XM_013855840.2	5′-GATCCTGCGGATGAGGATAA-3′	5′-AAGCAGCTTGTCATCCGAGT-3′
BnSUT2	EF613226	5′-TGAGAACATTGCCAAGCAAG-3′	5′-CAACAGCAAAATGGAGAGCA-3′
BnSUT4	XM013787637	5′-AAGAAGCTTCCACGTCCAGA-3′	5′-TCTATGCCATTGCCAACGTA-3′
BnSWEET11	XM_013871605.2	5′-AGGTGACCCTCGATATGCTG-3′	5′-TCATGTAGCTGTTGCGGAAG-3′
Actin	AF111812.1	5′-GATTCCGTTGCCCTGAAGTA-3′	5′-GCGACCACCTTGATCTTCAT-3′
